# A model of the natural history of screen-detected prostate cancer, and the effect of radical treatment on overall survival

**DOI:** 10.1038/sj.bjc.6603105

**Published:** 2006-04-25

**Authors:** C Parker, D Muston, J Melia, S Moss, D Dearnaley

**Affiliations:** 1 Academic Unit of Radiotherapy & Oncology, Institute of Cancer Research and Royal Marsden NHS Foundation Trust, Downs Road, Sutton SM2 5PT, UK; 2 Cancer Screening Evaluation Unit, Institute of Cancer Research, Downs Road, Sutton SM2 5PT, UK

**Keywords:** prostate cancer, model, outcomes

## Abstract

The lead time and overdetection associated with prostate-specific antigen (PSA) screening, and generational improvements in all-cause mortality, make prostate cancer outcome studies from the pre-PSA era difficult to interpret in a contemporary setting. We developed a competing-risks hazard model to estimate the natural history of screen-detected prostate cancer, and the impact of radical treatment on overall survival. The model of hazard of mortality was fitted to clinical outcome data from the pre-PSA era, and the effects of screening, generational mortality improvements and radical treatment were incorporated. Sensitivities to the choice of baseline data and values of key parameters were assessed. Lead-time estimates in men diagnosed aged 55–59 years were 14.1, 9.3 and 5.0 years for men with Gleason scores <7, 7 and >7, respectively, assuming biennial screening with 100% attendance. Central estimates of 15-year prostate cancer mortality for conservative management of screen-detected prostate cancer ranged from 0 to 2% for Gleason scores <7, 9 to 31% for Gleason score 7 and 28–72% for Gleason scores >7. For men aged 55–59 years at diagnosis, the predicted absolute 15-year survival benefit from curative treatment was 0, 12 and 26% for men with Gleason scores <7, 7 and >7, respectively. Estimates of the survival benefit of radical treatment were relatively insensitive to values of key parameters. The case for curative treatment, rather than conservative management, of screen-detected localised prostate cancer is strongest in men with high-grade disease. This conclusion contrasts with current patterns of care.

For most invasive cancers, effective curative treatment makes all the difference between life and death. While prostate cancer can follow an aggressive and ultimately fatal course, similar to that of other cancers, a significant proportion of cases will behave in an indolent fashion, with no impact either on health or longevity, even in the absence of treatment. This effect is compounded by competing causes of mortality in the typical age distribution affected by prostate cancer. Partly for these reasons, it has been difficult to establish whether radical treatment of prostate cancer improves overall survival, and, if so, by how much.

Retrospective comparisons of curative treatment *vs* conservative management of prostate cancer are of limited value, given the likelihood of significant confounding variables. There are only two published randomised controlled trials comparing expectant management and definitive treatment of prostate cancer. Of these, one, which included just 142 patients, is too small for meaningful analysis (Iversen *et al*, 1995). The other, the Scandinavian Prostatic Cancer Group Study Number 4, which opened in 1989, randomised 695 men with localised disease between radical prostatectomy and watchful waiting (Bill-Axelson *et al*, 2005). Patients were aged less than 75 years, with well or moderately differentiated, clinically localised prostate cancer, a prostate-specific antigen (PSA) of less than 50 ng ml^−1^ and a life expectancy of at least 10 years. At a median follow-up of 8.2 years, randomisation to radical prostatectomy was associated with improved disease-specific mortality, with a hazard ratio of 0.56 (95% CI: 0.36–0.88; *P*=0.01), which translated into an absolute overall survival benefit of 5.3% (95% CI: −0.3 to 11.0%) at 10 years. Although the Scandinavian trial provides the best available data comparing conservative management and curative treatment, there are very major differences between Scandinavian practice during the early 1990s and contemporary clinical practice. In particular, the case-mix in the trial was typical of clinically detected prostate cancer from the pre-PSA era, with only 12% of patients having stage T1c disease. It is therefore unclear how the results should be applied to men with screen-detected prostate cancer.

Prostate-specific antigen screening results in overdetection (of cases that would not otherwise have been detected) and introduces a lead time (the time difference between screen detection and clinical detection in the absence of screening), which may be of the order of 10 years or more (Draisma *et al*, 2003). It follows that, in the absence of treatment, the natural history of screen-detected prostate cancer will appear more favourable than that of clinically detected prostate cancer from the pre-PSA era. This is an important consideration for men faced with the choice between conservative management and curative treatment. In comparison with clinically detected disease, men with screen-detected cancers will have longer to endure any adverse effects of curative treatment, and longer to wait for any beneficial effect on survival to emerge.

There are two ongoing randomised trials that aim to compare curative treatment with conservative management in PSA screen-detected disease. The Prostate Cancer Intervention *v**ersus* Observation Trial (PIVOT) compares radical prostatectomy *vs* watchful waiting, and is now closed to recruitment with 731 patients entered (Wilt and Brawer, 1995). The Prostate testing for cancer and Treatment (ProtecT) study compares radical prostatectomy, external beam radiotherapy and active monitoring, and aims to recruit over 2000 patients (Donovan *et al*, 2003). The long-term mortality outcomes from PIVOT and the ProtecT trial will provide the best data concerning the effectiveness of curative treatment of screen-detected disease, but will not be available for some years. In the meantime, knowledge of the potential impact of treatment on survival would be of great value to men found to have screen-detected prostate cancer, and who are faced with the decision whether or not to have curative treatment. We have therefore undertaken a modelling exercise to estimate the natural history of screen-detected prostate cancer, and the impact of curative treatment on overall survival.

## METHODS

**Figure 1:**
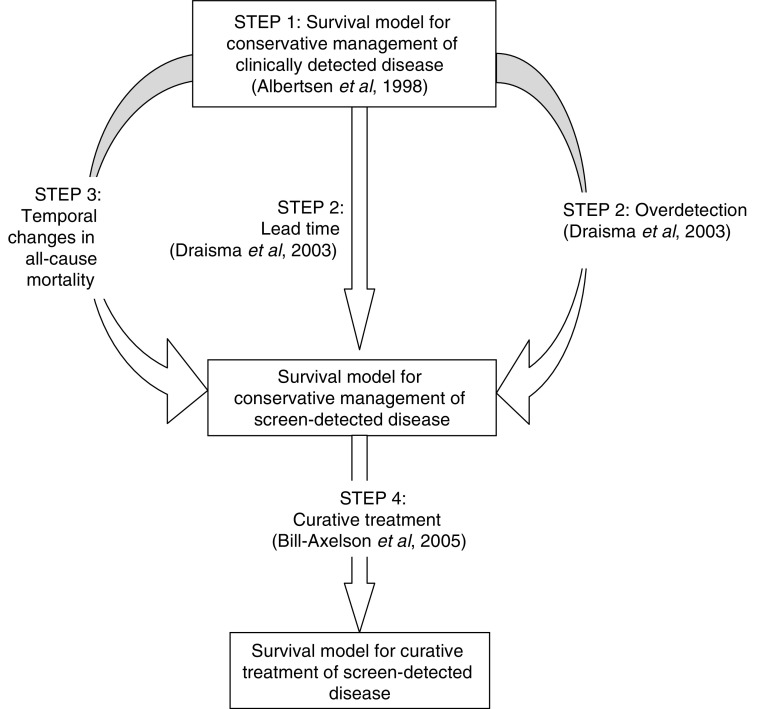
Overview of methods.

We constructed a competing-risks model of hazard from mortality to describe the natural history of prostate cancer from the pre-PSA era, using terminology and results outlined in Anderson *et al* (2002) (step one). We then adapted this model to incorporate the lead time and the probability of overdetection associated with PSA screening (step two), and generational improvements in all-cause mortality (step three). We used this model, together with evidence from the Scandinavian Prostatic Cancer Group Study (Bill-Axelson *et al*, 2005), to make projections of the effect of curative treatment on overall survival in contemporary patients with screen-detected localised prostate cancer (step four). This approach is summarised in Figure 1.

### Step one: constructing the competing risks model

The hazard due to other-cause mortality under the given treatment was allowed to vary with time by assuming a Weibull survival distribution, with scale parameter *λ*_O_ and shape parameter *γ*: *h*_O_(*t*)=*λ*_O_ *γ t*^*γ*−1^. Disease-specific mortality was assumed, for simplicity, to provide a constant hazard and therefore an exponential survival distribution, with single parameter *λ*_P_: *h*_P_(*t*)=*λ*_P_, where *t* represents time since diagnosis. The total hazard is the sum of the disease-specific and other-cause hazards: *h*(*t*)=*h*_P_(*t*)+*h*_O_(*t*). The model therefore requires estimates of three model parameters.

Our model parameterisation does not directly account for age and Gleason score, which are important covariates in modelling survival from prostate cancer, so it was necessary to stratify the model by these variables. To fit the model, we therefore required long-term survival outcome data from conservative management of prostate cancer, stratified by age group and Gleason score. Average or smoothed proportions (so as to eliminate random within-group variation) of the cohort alive, dead due to prostate cancer and dead due to other causes at a given time point would be sufficient to calculate two of the three parameters in the model, conditional on a value for the other.

We reviewed the literature systematically for research that would provide the necessary cohort survival data. Only one source was found: Albertsen *et al* (1998), 2005 monitored the survival of 767 men from the Connecticut Tumor Registry with localised prostate cancer diagnosed between 1971 and 1984, treated conservatively and followed up for a median of 24 years. Long-term survival probabilities fitted by proportional hazards statistical methods (Poisson regression over 15 years) were provided (Table 3 of (Albertsen *et al*, 1998)), stratified by age at diagnosis (within 5 years) and biopsy Gleason score (2–4, 5, 6, 7, 8–10).

Parameter *γ* controls the rate of increase of other-cause hazard over time rather than, directly, the level of a hazard, so is of less interest than other parameters. We therefore decided to condition our estimates for *λ*_O_ and *λ*_P_ on a value for *γ*. The value for *γ* was estimated by fitting a single-risk Weibull model to 10 000 survival periods simulated from period analyses of three US life tables (9–11), with initial ages sampled uniformly between 55 and 74 years inclusive. Model A assumed constant scale and shape parameters across all age groups, model B additionally assumed interaction between the scale parameter and age group and model C additionally assumed interaction between the shape parameter and age group. Fitted parameter values are shown in Table 1.

Being period life tables, it is uncertain how fitted parameter values might relate to survival in a cohort. However, a single value of 1.8 was chosen for the shape parameter *γ* as fitted values for the shape parameter are close to this value in both 1979–1981 and 1989–1991 life tables, which cover periods close to those over which the Albertsen study (Albertsen *et al*, 1998, 2005) was conducted.

We calculated *λ*_O_ and *λ*_P_, conditional on *γ*=1.8, for each stratum using an iterative numerical algorithm written specifically for the task in Stata version 8.2 (Stata Corporation, College Station, TX, USA), the software used throughout this paper. The model relies in part on a reliable estimate of the shape parameter *γ*. The main end point, projected probability of survival at 15 years, however, appears largely insensitive to small variations in this parameter. Given a value for the parameter (*γ*=1.8), values of *λ*_O_ and *λ*_P_ fitted to the data are detailed in Table 2.

### Step two: adapting the model to allow for PSA screening

We adapted the model to allow for the lead time introduced by PSA screening and the probability of overdetection. There are no published data providing lead times and overdetection rates by Gleason score and by age at diagnosis. The lead-time values and overdetection proportions used for the model were taken as average values simulated by a replica of the Markov lead-time model fitted by Draisma *et al* (2003) for ‘relevant’ cases (detections that were not overdetections), stratified by age (within 5 years) and biopsy Gleason score (<7, 7, >7), assuming screening with 100% attendance every 2 years. The replica model was tested to assess how close its predictions of lead times and overdetection for various screening programmes were to those quoted from the original model (Draisma *et al*, 2003).

We averaged the fitted 15-year survival results from Albertsen *et al* (1998) in the Gleason score 2–4, 5 and 6 groups, weighted according to their sample size over those categories. By doing this, Gleason score categories in our model corresponded with those in Draisma *et al* (2003).

The survival outcomes for the overdetected proportion of cases were calculated assuming *h*_P_(*t*) was equal to zero; for the ‘relevant’ cases, we calculated survival outcomes by setting *h*_P_(*t*) to be zero for time *t* less than the lead time, and equal to *λ*_P_ otherwise. For both groups, *h*_O_(*t*) was left unchanged in this step by using the parameter values fitted in step one. Overall survival outcomes for screen-detected cases were calculated as the average of the two sets of survival outcomes figures, weighted according to the probability of being in each group.

### Step three: updating the model to a contemporary population

Evidence of considerable reductions in mortality rates over the last 20 years can be found in US life tables (US Department of Health National Center for Health Statistics and Human Services). In fact, mortality at each age in every G7 country (Canada, France, Germany, Italy, Japan, UK, US) has declined exponentially in the last five decades at a roughly constant rate (Tuljapurkar *et al*, 2000). We accounted for mortality improvements in our modelling by multiplying fitted values of *λ*_O_ and *γ*, the two parameters in the other-cause mortality function, by values informed through the single-risk Weibull modelling of simulated life table survival data. In the central projections, the multipliers used were 0.25 and 1.15, respectively, chosen through consideration of the ratios shown in the final two columns of Table 1.

### Step four: using the model to assess treatment benefit

In order to assess the benefit of curative as opposed to conservative treatment, we required a model for mortality following curative treatment. The Scandinavian Prostatic Cancer Group Study assume in their analysis disease-specific hazards are proportional in relation to treatment (Bill-Axelson *et al*, 2005), an assumption we continued to adopt. We used their central estimate of the hazard ratio, 0.56, as the value for our parameter *HR*. We assumed a hazard function due to disease-specific mortality such that the disease-specific hazard ratio was equal to the parameter *HR* for all age group and Gleason score strata at all times. The form of the hazard function *h*_Q_(*t*) and its derivation are described in the Appendix A.

### Sensitivity analysis

To estimate the sensitivity of our results to the choice of baseline treatment/data set, the procedure described above was repeated for summary data from a Mayo Clinic study by Sweat *et al* (2002) of the long-term survival with prostate cancer of 751 men after radical prostatectomy, the only research found in the literature providing the necessary cohort survival data in curatively treated men. The same value of *γ* was used as before. New values of *λ*_O_ and *λ*_P_ were calculated based on reported fitted survival probabilities by age group and Gleason score strata at 20 years using Cox proportional hazards regression. As the base data relates to a cohort given curative rather than conservative treatment, *h*_P_(*t*) was the disease-specific hazard function under curative treatment and *h*_Q_(*t*) the disease-specific hazard function under conservative treatment with *HR* now equal to 1.79 (=1/0.56).

To assess the sensitivity of results to key parameters, we have also projected survival under ‘low’ and ‘high’ treatment benefit scenarios. Relative to the central scenario, the ‘low treatment benefit’ scenario assumes a higher overall survival hazard ratio for curative treatment (0.7), a lower improvement over time in other cause mortality (*λ*_o_ multiplier of 0.7 and *γ* multiplier of 1.3), longer lead times and greater overdetection rates; the ‘high treatment benefit’ scenario assumes a lower hazard ratio (0.45), higher other-cause mortality improvement (*λ*_o_ multiplier of 0.1 and *γ* multiplier of 1.0), shorter lead times and lower overdetection rates. The sensitivity ranges for the hazard ratio, lead time and overdetection rate were set as one standard deviation either side of the central estimate. In doing this, we assumed lead times, the logarithm of the hazard ratio and the logit of the overdetection rates were distributed normally.

## RESULTS

### Constructing the model

Average lead times and overdetection rates by age at diagnosis and by Gleason score from simulations of 1 million men with prostate cancer by the replica of Draisma's model (Draisma *et al*, 2003) for biennial screening are shown in Table 4.

The discrepancies between average lead times presented from Draisma's original model (Draisma *et al*, 2003) and our replica were found to be small: in the simulations, there was no more difference in the projected mean lead times for all cases and relevant cases only (cases of prostate cancer that would have been clinically detected) than 0.3 years for single screens, 0.2 years for schedules of annual screening and 0.3 years for schedules of quadrennial screening. The proportions of detections that were not ‘relevant’ agreed within 4% for all screening programmes.

For biennial PSA screening, the predicted average lead times for relevant cases were 9.9–14.1 years for cases with Gleason scores <7, 8.0–9.3 years for those with Gleason score 7 and 5.0–6.0 years for Gleason scores >7.

### Projection results

**Figure 2:**
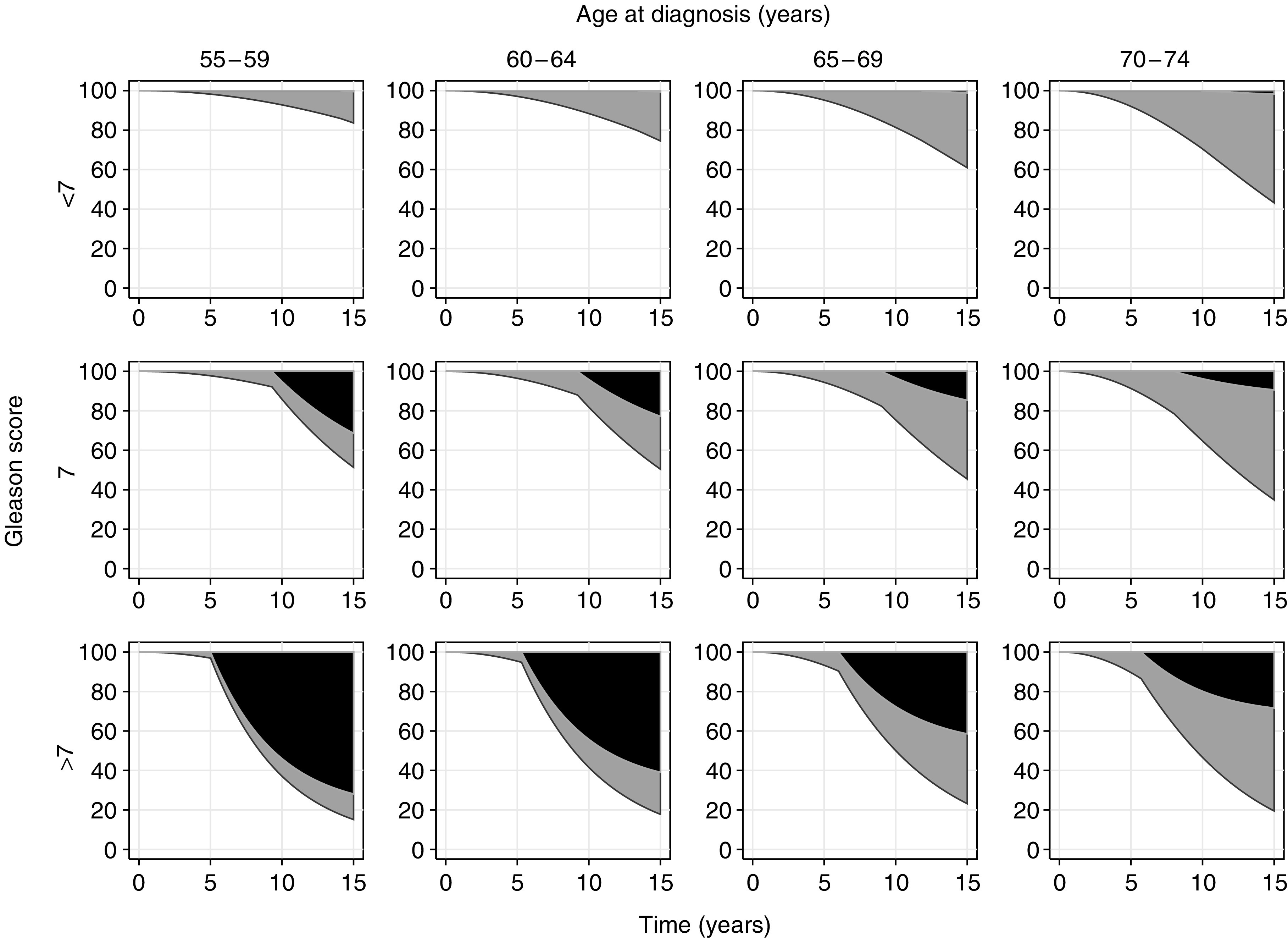
Projections of survival over 15 years under conservative treatment, using the central model assumptions. Black areas represent deaths due to prostate cancer, grey areas represent deaths due to other causes.

Figure 2 shows projected survival over 15 years for contemporary patients with PSA screen-diagnosed prostate cancer managed conservatively, using the central model assumptions (based on data from Albertsen *et al* (1998), with 100% attendance at biennial screening and multipliers for *λ*_O_ and *γ* of 0.25 and 1.15, respectively). Mortality is divided by projected cause (prostate cancer *vs* other). Table 3 provides survival projections at 15 years – sections (i)–(iii) of the table relate to the natural history of prostate cancer in conservatively treated men and section (iv) relates to survival in curatively treated men. The projected treatment effect, in terms of the absolute difference in 15-year survival, is shown in section (v).

Under the central assumptions based on conservative management, the probability of 15-year mortality from prostate cancer appears strongly dependent on grade, particularly so for men who are younger at diagnosis. For example, for men aged 55–59 years at diagnosis, the projected 15-year prostate cancer mortality is 0%, 31% and 72% for men with Gleason scores <7, 7 and >7, respectively. For men aged 70–74 years at diagnosis, the 15-year prostate cancer mortality is projected to be 2%, 9% and 28% for men with Gleason scores <7, 7 and >7, respectively.

The projected overall survival benefit from curative treatment appears similarly dependent on Gleason score and age at diagnosis. For example, for men aged 55–59 years at diagnosis, the absolute 15-year survival benefit from curative treatment is 0%, 12% and 26% for men with Gleason scores <7, 7 and >7, respectively. For men aged 70–74 years at diagnosis, the 15-year overall survival benefit is projected to be 1%, 3% and 6% for men with Gleason scores <7, 7 and >7, respectively.

### Sensitivity analyses

Central projections of long-term survival probabilities together with the range of outcomes projected from the high and low scenarios using baseline data from either Albertsen *et al* (1998) or Sweat *et al* (2002) are shown in Table 4 with the ranges of lead times and overdetection rates assumed. While survival probabilities based on data from Sweat are generally higher than those based on data from Albertsen, likely due to differences in the case-mix of the two studies, the point estimates are reasonably close and there is considerable overlap in the range of projected survival probabilities and absolute benefits, suggesting little sensitivity to baseline data set. Ranges of survival projections across scenarios are wide, but ranges of estimates of the absolute benefit due to treatment are small, suggesting little sensitivity of absolute benefit estimates to key parameter values. For example, the 15-year absolute benefit of curative treatment to a man diagnosed at age 60–64 years with a Gleason 7 cancer is 9% (range across three scenarios: 0–32%) based on Albertsen's data (Albertsen *et al*, 1998) and 4% (range: 0–18%) based on Sweat's data (Sweat *et al*, 2002).

## DISCUSSION

This is the first study, to our knowledge, to model both the natural history of screen-detected prostate cancer and the impact of radical treatment on overall survival. While the outputs of the model must be interpreted with caution, they may have important implications for targeting treatment to those patients who stand to benefit most. Specifically, the absolute survival benefit of radical treatment is predicted to be greater in men with high-grade disease, whereas current clinical practice preferentially targets radical treatment to patients with low-grade prostate cancer.

The central projections of the model based on Albertsen's data (Albertsen *et al*, 1998) are that the 15-year prostate-cancer-specific mortality for men aged 55–74 years diagnosed with screen-detected prostate cancer with a Gleason score of <7, and managed conservatively, will be 1%, and the absolute benefit in 15-year overall survival from curative treatment of such cases is predicted to be less than 1%. The decision whether or not to have radical treatment is a value judgement, comparing the known morbidity of treatment with the potential survival benefit. Faced with a 30–60% risk of treatment-related impotence, and a 0–1% 15-year survival benefit, the majority of patients would decline radical treatment (Singer *et al*, 1991). In fact, data from the Cancer of the Prostate Strategic Urologic Research Endeavor (CaPSURE), an observational database of prostate cancer patients from 35 US centres, on 2078 cases diagnosed between 1989 and 2001 with low-risk prostate cancer (serum PSA </=10 ng ml^−1^, Gleason sum </=6 and clinical *T* stage </=T2a), show that just 7.9% of such men chose an observation policy, rather than treatment (Cooperberg *et al*, 2004). One explanation for this pattern of care is that patients and their clinicians have unrealistically high expectations of the survival benefit from treatment of low-risk disease, and this possibility deserves further study.

The model predicts that the absolute survival benefit of radical treatment for screen-detected prostate cancer is greater in higher-grade disease. This finding is consistent with observations from retrospective studies. A population-based study from the SEER database, based on data for 59 876 cancer-registry patients aged 50–79 years who had clinically localised prostate cancer diagnosed between 1982 and 1992, analysed disease-specific survival by grade and by type of treatment (Lu-Yao and Yao, 1997) and found the absolute difference in 10-year disease-specific survival between radical prostatectomy and watchful waiting was 1, 10 and 22% for grade 1, 2 and 3 disease, respectively. Similarly, a multicentre comparison based on over 2000 patients, diagnosed between 1971 and 1984, observed a 10-year disease-specific survival difference between radical prostatectomy and expectant management of 4% for cases with Gleason score 2–4, 8% for scores 5–6, 17% for scores of 7 and 19% for scores 8–10 (Barry *et al*, 2001). Patients who select watchful waiting are very different from those who select radical prostatectomy, and so retrospective comparisons between them should be interpreted with great caution. However, it is plausible that the differences between these two groups of patients are independent of tumour grade. So, taken together with the results of these retrospective studies, our model supports the hypothesis that the absolute survival benefit of radical treatment is greater for high-grade disease. These observations contrast with current patterns of care for men with localised prostate cancer. Of patients on the CaPSURE database with localised disease diagnosed from 1999 to 2001, primary treatment was with radical intent in 77.5, 75.8 and 47% of low-, intermediate- and high-risk cases, respectively (Cooperberg *et al*, 2003).

The majority of prostate cancers detected by PSA screening are low grade. Data on the grade-mix of screen-detected cancers from second round and subsequent screens shows 76–85% of cases with Gleason scores <7, 13–20% with a Gleason score of 7 and 1–4% with Gleason scores >7 (Hoedemaeker *et al*, 2001; Hugosson *et al*, 2004; Makinen *et al*, 2004). Based on these figures, and given the median age of detection of 67 years, our model predicts that the absolute 15-year overall survival benefit for radical treatment, compared with conservative management, of all screen-detected localised prostate cancers will be approximately 1–2%. It is noteworthy that the ongoing clinical trials addressing this issue (PIVOT and ProtecT) are not adequately powered to detect a survival benefit of this magnitude.

Nicholson and Harland (2002) have previously modelled the natural history of screen-detected prostate cancer, based on temporal trends in prostate cancer incidence and mortality at a population level. They did not include consideration of the effect of age and grade on lead times, generational improvements in all-cause mortality or the impact of treatment on prostate cancer outcomes. Notwithstanding these methodological differences, their projections of 15-year disease-specific mortality for screen-detected cancers diagnosed between ages 55 and 74 years, and managed conservatively, ranged from 7.4 to 11.6%, which are comparable to the current study.

The accuracy of our model is dependent on the validity of the underlying assumptions. To model the natural history of PSA screen-detected cancers, we have used published data to derive estimates, by patient age and Gleason score, for survival outcomes from expectant management in the pre-PSA era, and for lead times and overdetection rates associated with PSA screening. The Albertsen series (1, 8) used to describe the outcome of conservative management of localised disease from the pre-PSA era is highly regarded on account of its size, the maturity of the outcome data and the use of centralised pathology review with the Gleason scoring system. However, 70% of patients in that series did not have a staging bone scan, and there is evidence of systematic upgrading in the interpretation of the Gleason grading system over time (Smith *et al*, 2002; Kondylis *et al*, 2003). For both these reasons, the data from Albertsen are likely an overestimate, rather than an underestimate, of ‘true’ prostate cancer mortality from the pre-PSA era. In any event, using the Mayo Clinic radical prostatectomy data (Sweat *et al*, 2002), to describe the outcome of prostate cancer from the pre-PSA era, does not materially affect the outputs of the model. The model for estimating mean lead times and overdetection rates was derived from MIcrosimulation SCreening ANalysis (MISCAN) simulations of results validated against data from the Rotterdam section of the European Randomised Study of Screening for Prostate Cancer (ERSPC), which enrolled 21 166 men in the unscreened arm and 21 210 men in the screened arm and in which 1498 prostate cancers were diagnosed. The Rotterdam screening policy evolved during the study, but predominantly consisted of a sextant prostate biopsy for men with a PSA >3 ng ml^−1^. MISCAN models are based on Markov processes of states and transitions and are designed to evaluate cancer-screening programmes.

To model the absolute survival benefit of curative treatment in the context of PSA screen detection, we used data from the only randomised trial available, the Scandinavian Prostatic Cancer Group Study. The hazard ratio for the effect of radical treatment on prostate cancer mortality was taken to be 0.56 (95% CI: 0.36–0.88), and this hazard ratio was assumed to be independent of tumour grade and patient age. Subgroup analyses of the Scandinavian trial are consistent with these assumptions (Bill-Axelson *et al*, 2005). However, the trial consisted predominantly of patients with low-grade disease, and included just 159 cases (23%) with Gleason score 7, and 35 cases (5%) with Gleason score >7. A further assumption is that the hazard ratio for the benefit of curative treatment on prostate cancer mortality, derived from data on patients with cancers typical of the pre-PSA era, is applicable to patients with screen-detected cancers. In the absence of any data on this issue, this is a significant limitation.

## CONCLUSIONS

This modelling exercise, based on published data, describes the natural history of screen-detected prostate cancer and the impact of radical treatment on overall survival. The results of the model should be interpreted with caution, since the original data upon which it is based cannot necessarily be assumed to be generalisable more widely. With that proviso, the 15-year mortality from low-grade, screen-detected prostate cancer in men aged 55–74 years at diagnosis, who elect conservative management, is projected in our central model to be 1%, and the absolute 15-year survival benefit of curative treatment, less than 1%. The absolute survival benefit for radical treatment is predicted to be greater in men with high-grade disease. When the results of PIVOT and the ProtecT trial are mature, subgroup analysis by grade will be important. Until then, the predictions of our model, together with the available retrospective data, suggest that the case for radical treatment, rather than conservative management, of localised prostate cancer is strongest in men with high-grade disease.

## Figures and Tables

**Table 1:** Scale (× 100) and shape parameters fitted by the Weibull model of simulated survival from a period analysis of US life tables

**Starting age group (years)**	**Parameter**	**1979–1981**	**1989–1991**	**2002**	**Ratio 1989–1991 : 1979–1981**	**Ratio 2002 : 1979–1981**
55–59	Scale × 100	0.25	0.17	0.07	0.66	0.28
60–64	Scale × 100	0.79	0.47	0.20	0.59	0.26
65–69	Scale × 100	2.84	1.54	0.70	0.54	0.25
70–74	Scale × 100	11.02	4.47	2.17	0.41	0.20
						
55–59	Shape	2.01	2.09	2.30	1.04	1.14
60–64	Shape	1.81	1.93	2.12	1.06	1.17
65–69	Shape	1.63	1.76	1.93	1.08	1.18
70–74	Shape	1.50	1.70	1.79	1.14	1.19

**Table 2:** Fitted values to the basic model from Albertsen's data of parameters 100 × *λ*_O_ (‘other’) and 100 × *λ*_P_ (‘prostate’) given *γ*=1.8

	**Age at diagnosis (years)**							
	**55–59**		**60–64**		**65–69**		**70–74**	
**Gleason score**	**Other**	**Prostate**	**Other**	**Prostate**	**Other**	**Prostate**	**Other**	**Prostate**
<7	0.25	0.99	0.42	1.17	0.70	1.74	1.19	2.63
7	0.32	9.84	0.52	8.62	0.82	7.58	1.31	6.37
>7	0.45	19.53	0.68	17.50	0.99	14.75	1.58	12.39

*λ*_O_=scale parameter; *γ*=shape parameter.

**Table 3:** Central projections of 15-year outcome probabilities and absolute benefit of treatment (to nearest whole percentage point). (‘PC death’: death attributed to prostate cancer)

	**Age at diagnosis (years)**											
	**55–59**			**60–64**			**65–69**			**70–74**		
**Gleason score**	**Alive**	**Other death**	**PC death**	**Alive**	**Other death**	**PC death**	**Alive**	**Other death**	**PC death**	**Alive**	**Other death**	**PC death**
*(i) Original data – conservative treatment*												
<7	62	26	12	48	38	13	31	52	17	14	64	22
=7	15	15	70	14	24	62	11	36	53	7	51	42
>7	3	10	87	3	16	81	3	25	72	2	38	60
												
*(ii) Conservative treatment, adjusting for screening (step two)*												
<7	71	28	1	57	42	1	39	60	1	20	79	1
=7	42	31	27	37	45	18	27	63	10	15	79	6
>7	11	25	64	11	38	51	12	57	31	7	75	18
												
*(iii) Conservative treatment, allowing for screening and contemporary population (step three)*												
<7	84	16	0	74	25	1	61	38	1	43	55	2
=7	52	17	31	50	27	23	45	40	15	35	56	9
>7	15	13	72	18	21	61	23	35	42	20	52	28
												
*(iv) Curative treatment, allowing for screening and contemporary population (step four)*												
<7	84	16	0	75	25	0	61	38	1	44	55	1
=7	64	18	18	59	28	13	51	41	8	38	57	5
>7	41	19	40	38	28	34	35	42	23	26	58	16
												
*(v) Absolute differences: (iv) minus (iii)*												
<7	0	0	0	1	0	−1	0	0	0	1	0	−1
=7	12	1	−13	9	1	−10	6	1	−7	3	1	−4
>7	26	6	−32	20	7	−27	12	7	−19	6	6	−12

**Table 4:** Projections of 15-year survival probabilities based on data from Albertsen *et al* (1998) and Sweat *et al* (2002): central estimates and ranges of values obtained from low and high projections of life expectancy

		**Lead time (years)**		**Overdetection rate (%)**		**Albertsen data: survival benefit (%)**		**Sweat data: survival benefit (%)**	
**Age group (years)**	**Gleason score**	**Central**	**Sensitivity range**	**Central**	**Sensitivity range**	**Central**	**Sensitivity range**	**Central**	**Sensitivity range**
55–59	<7	14.1	5.2–22.1	37.1	6.9–82.4	0	0–5	0	0–6
	=7	9.3	2.8–15.1	16.1	1.2–74.5	12	0–36	5	0–17
	>7	5.0	1.1–9.6	7.3	0.2–78.6	26	1–48	12	0–23
									
60–64	<7	13.4	5.1–21.7	54.1	13.7–89.8	1	0–5	0	0–6
	=7	9.2	2.7–15.3	27.8	4–78.2	9	0–32	4	0–18
	>7	5.3	1.2–9.7	12.1	0.6–74.7	20	0–46	11	0–24
									
65–69	<7	11.8	4.4–20.1	66.9	19.4–94.4	0	0–7	0	0–4
	=7	9.0	2.7–15.2	43.5	9.3–85.3	6	0–27	3	0–15
	>7	6.0	1.4–10.1	25.8	3.4–77.4	12	0–40	6	0–22
									
70–74	<7	9.9	3.4–17.8	76.6	23.6–97.2	1	0–9	0	0–4
	=7	8.0	2.3–14.3	57.5	15.2–91.1	3	0–22	2	0–15
	>7	5.7	1.7–10.6	36.9	6.9–82.3	6	0–34	5	0–20

## Appendix A

Marginal survival from death due to prostate cancer to time *t* since diagnosis under conservative treatment is *S*_P_(*t*)=exp(−*λ*_P_ *t*). Given a hazard ratio of *HR*, survival from death due to prostate cancer to time *t* since diagnosis under curative treatment is *S*_Q_(*t*)=1−*HR* × [1−exp(−*λ*_*P*_ *t*)]. Noting that (Anderson *et al*, 2002) *h*_Q_(*t*)=−d/d*t* log *S*_Q_(*t*), we obtain *h*_Q_(*t*)={*HR* *λ*_P_ exp(−*λ*_P_ *t*)}/{1−*HR* × [1−exp(−*λ*_*P*_ *t*)]}.
